# The role of surgical timing in survival of patients with spinal metastases

**DOI:** 10.1007/s10147-026-03099-8

**Published:** 2026-06-16

**Authors:** Miklós Kopa, Ádám Kővári, Lukács Németh, János Báskay, Péter Pollner, Zoltán Nagy, Péter Banczerowski, Tamás Mezei

**Affiliations:** 1 Department of Neurosurgery, Semmelweis University Hungary, Amerikai út 57, Budapest, 1145 Hungary; 2 Clinic of Neurosurgery and Neurointervention, Semmelweis University Hungary, Amerikai út 57, Budapest, 1145 Hungary; 3 Health Data Science and AI Knowledge Centre, Health Services Management Training Centre, Faculty of Health and Public Administration, Semmelweis University Hungary, Kútvölgyi út 2, Budapest, 1125 Hungary; 4 Department of Biological Physics, Eötvös Loránd University Hungary, Pázmány Péter sétány 1/a, Budapest, 1117 Hungary

**Keywords:** Spinal metastases, Prognosis, Survival, Surgical treatment, Operation timing

## Abstract

**Background:**

Spinal metastases commonly cause pain, neurological deterioration, and instability. Surgery is palliative, aiming to relieve symptoms and maintain oncologic treatment eligibility. Optimal surgical timing, however, remains debated.

**Methods:**

We retrospectively analyzed 664 patients who underwent 760 operations for spinal metastases at Semmelweis University (2008–2022). Clinical data included demographics, comorbidities, primary tumor, neurological status, surgical approach, and timing of surgery relative to the symptom onset. Survival was assessed with both Kaplan–Meier and log-rank testing, with subgroup analyses by surgical timing and operative hours.

**Results:**

Pain (83%) and motor deficits (46%) were the most frequent symptoms. Lung, renal, breast, and hematologic cancers predominated. Median survival was 294 days; 30% exceeded two years. No survival advantage was detected for conventional acute surgical thresholds (≤ 48 h, ≤ 72 h, ≤ 1 week). Delays beyond one week correlated with reduced survival. Pain improved postoperatively in ~ 90% of cases, independent of timing. Severe preoperative motor dysfunction predicted poor survival and recovery, emphasizing the need for urgent surgery in this subgroup. Procedures during working hours were associated with superior postoperative symptom control (OR 3.3, *p* = 0.003), though survival outcomes were unchanged.

**Conclusions:**

Surgical management of spinal metastases provides consistent pain relief. Urgent intervention is critical in patients with progressive motor deficits, whereas multidisciplinary preoperative optimization is preferable in others. Operations during working hours yield better symptomatic outcomes, but survival remains primarily determined by the oncologic disease burden. Prospective multicenter studies are warranted to refine timing guidelines.

## Introduction

The incidence of cancer is increasing year by year, with almost 20 million new cancer patients diagnosed worldwide annually; based on the data of the Global Cancer Observatory, an estimated number of 10 million patients will die every year in the coming decades [1]. Consequently, cancer remains one of the leading causes of decreased life expectancy globally. In the 21st century cancer imposes a substantial burden on the health care worldwide, by being among the 3 major causes of death in 177 of 183 countries [2]. Primary tumors from any organ can metastasize to the spine, most commonly prostate, breast and lung cancers [3]. Nearly 70% of cancer patients develop spinal metastases throughout their lives, of which 10% cause compression of the spinal cord [4].

Spinal tumors can be divided into 3 subgroups, based on anatomical localization: (1) intramedullary, (2) intradural-extramedullary, (3) extradural [5]. The most common extradural spinal tumors are metastases [6]. Symptoms caused by spinal tumors can be diverse, depending on the location, size, and growth pattern; however, the main leading symptom is pain, which is present in almost 95% of the cases [7]. Metastatic spinal cord compression (MSCC) causes intractable pain, disability, and incontinence, which severely impairs quality of life in the terminal stage of the disease [8].

The treatment of spinal metastases requires a multidisciplinary approach to ensure personalized care [9]. These patients often have a poor prognosis, and surgical treatment is primarily palliative, aiming to relieve pain, improve neurological function, and maintain eligibility for further oncological therapy [10]. While conservative treatments, including analgesics, glucocorticoids, and bisphosphonates, are first-line options [11], radiotherapy remains a key modality, particularly for radiosensitive tumors [12]. Surgery is indicated in cases of compression of the spinal cord, causing various neurological deficits, radicular irritation, or spinal instability, with the goal of tumor resection, spinal decompression, and stabilization to enhance quality of life, making the patient suitable for further oncological treatment, thereby potentially extending overall survival [13]. Until the publication of this article, there are still no clear evidence-based guidelines on surgical interventional timing for spinal metastases. The most recent guideline mentions that in the case of rapid deterioration of neurological functions in *incomplete* spinal cord injury, meaning diminished but preserved motor and sensory functions, with or without autonomic dysfunction, performing acute surgical intervention is suggested (within 48 h).

It is also mentioned that after 24 h of complete spinal cord injury (complete loss of motor and sensory function, accompanied by autonomic impairment), the prognosis of neurological restoration is poor; therefore, indication for surgery shift toward alleviating intractable pain, typically not in the hyperacute phase [14].

## Methods

### Patient population

A retrospective review was conducted of 664 patients who underwent 760 surgical procedures for spinal metastases at the Department of Neurosurgery, Semmelweis University, Budapest, Hungary, (formerly National Institute of Mental Health, Neurology and Neurosurgery, or National Institute of Clinical Neuroscience) between December 2007 and January 2022. Inclusion criteria comprised adult patients (≥ 18 years) with surgically treated vertebral metastases.

### Surgical techniques

Excisional procedures were defined as corpectomy/total tumor removal with posterior stabilization. Palliative procedures included posterior stabilization with decompression (laminectomy) and/or partial tumor removal.

### Data collection

Collected variables included demographics (age, sex), baseline performance status (Karnofsky Performance Scale), presenting symptoms in neurological function (pain, paresis – according to the worldwide used Lovett’s paresis scale, and autonomous dysfunction), metastatic burden (extraspinal osseous or visceral disease, primary tumor site, histology), elapsed time between symptomatic onset and surgical intervention (timeframe), surgical details (levels involved, procedure type, postoperative course, hospital stay), and survival. Patients were also scored using established prognostic systems: revised Tokuhashi (2005), Tomita, modified Bauer, and van der Linden [15–18].

### Statistical analysis

Descriptive statistics (mean, median, percentiles) summarized baseline characteristics. Associations between covariates and categorical outcomes were tested using Fisher’s exact test. Odds ratios (OR) were calculated where appropriate. Overall survival, measured from the day of surgery, was estimated with Kaplan–Meier analysis; differences between groups were assessed with the log-rank test. Cox proportional hazards and logistic-regression formula was used to examine the role of various factors (i.e.: age, sex, tumor biology, etc.) in terms of survival and overall surgical outcome. Survival time was censored at 24 months. Surgical outcomes were compared with binomial testing. Statistical significance was defined as *p* < 0.05. Analyses were performed with R software (version 3.4.4; R Foundation for Statistical Computing, Vienna, Austria).

## Results

### Descriptive statistics

In this retrospective study, a sample of tumor patent’s cases were analyzed fulfilling the criteria mentioned above. A total number of 664 patients and 760 cases met our inclusion criteria, consisting of 285 (43%) female and 379 (57%) male patients. The median age was 62 years, with the youngest patient at the age of 18 years, the oldest was 85 years of age.

In the population that has been studied, the most common primary tumor types were lung cancer (18.8%), tumors of unknown origin (14.3%), hematological malignancies (12.8%), renal cancer (11.4%), and breast cancer (10.7%). The average hospital stay was 8.1 days. The most frequently observed symptoms were pain syndrome and motor deficits. The mean preoperative Karnofsky score was 65.8 (median: 60).

The thoracic spine was the most frequently affected spinal segment, with metastases in 356 cases (58%). Additionally, 146 patients (20%) had visceral metastases, and 124 patients (17%) had other bone metastases. Single vertebral involvement was present in 381 cases (52%), whereas multiple (three or more) vertebral metastases were observed in 196 cases (27%). The mean Charlson Comorbidity Index was 8.2. The most common symptom was pain, which was present in 83% of the surveyed population. Among the 303 patients diagnosed with motor deficits, the paresis scale distribution was as follows (according to Lovett’s paresis scale): 3/5 in 119 patients (39%), 4/5 in 78 patients (26%), 2/5 in 66 patients (22%), 0/5 in 30 patients (10%), and 1/5 in 10 patients (3%).

### Survival data

Our analysis showed a median survival time of 294 days, the survival periods, that exceeded 2 years, were censored. Of the cases analyzed, the date of death was known for 529 patients (70%), while 231 cases (30%) were censored, meaning survival exceeded 2 years. In the studied population, the shortest survival time was 4 days, while the longest was 731 days.

### Survival period in terms of timeframes

**Fig. 1:**
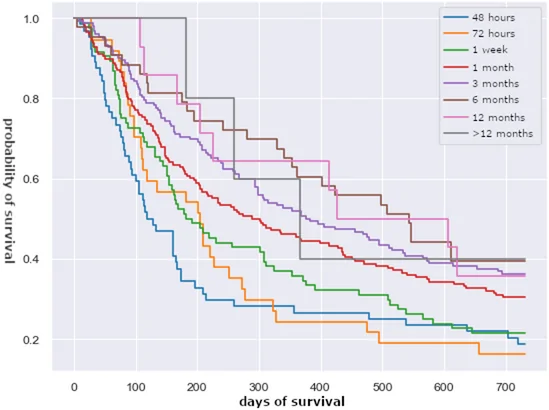
Survival period in terms of timeframes

Initially, the timeframes were categorized into 8 subgroups, and the survival periods were examined in each timeframes by using a Kaplan-Meier curve (Fig. 1). Because adjacent timeframes exhibited similar survival trajectories, they were consolidated into 3 clinically most relevant subgroups in terms of survival.

In examining the effects of surgical interventional timing on overall symptoms, survival and outcome, the widely used conventional 3 acute timeframes (elapsed time between the time of acute onset of the indicative symptom and the surgery) were used:

1. Within 48 h.
2. 48 to 72 h,
3. 72 h to 1 week.

**Fig. 2:**
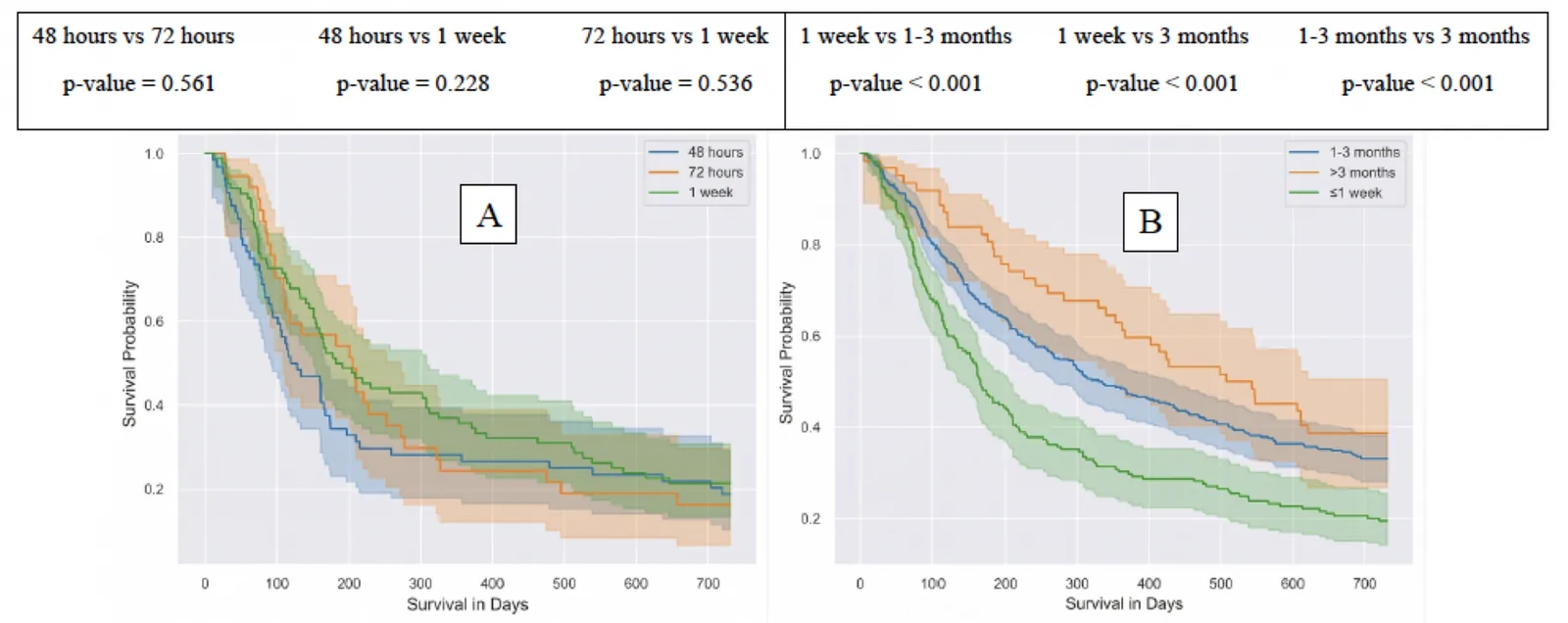
(**A**) Effects of conventional acute timeframe groups on survival. (**B**) Effects of new timeframe groups on survival

By evaluating these timeframes with Kaplan-Meier curves, surgeries performed within 1 week from symptomatic progression, no significant impact was found in aspects of survival (Fig. 2A)**.** However, widening the timeframes revealed significant differences in survival beyond the 1-week mark (Fig. 2B)**.**

### Evolution of symptoms regarding timeframes

The most observed symptoms were pain and paresis. These symptoms were significantly more common in the acute and subacute timeframed populations. Pain as a symptom was not further classified in the study. In the acute timeframed group (within 1 week), the presence of preoperative pain showed mostly equal distribution. In examining the postoperative changes - in the aspects of pain - in all timeframe groups, no significant correlation was found with Fisher’s tests between the timing of surgery and pain relief management, meaning that management of pain as a symptom is effective regardless of timeframes. When counting the case percentages, 90% symptomatic improvement was achieved.

By following the analysis with the new timeframes mentioned above, the study revealed a significant correlation between preoperative paresis and survival period, meaning motor function is a strong indicator for survival. Fisher’s test was used to confirm the probability of motor deficit presence in the acute timeframe, supporting the evidence that severe motor deficit is an indication for acute surgery (*p*-value = < 0.001, OR = 5.940).

The second most common symptom was motor dysfunction, which was divided into two categories, differing from international literature, but based on the relevance for surgical indication. The *mild motor dysfunction* group includes latent levels of weakness, specifically those scoring 5-4-3 on the paresis scale (Table 1). Analyzing these motor deficits across all timeframes by using Fisher’s test, we found that no significant difference was observed in postoperative improvement among patients operated within 1 week. The *severe motor dysfunction* group includes scores 2-1-0 on the paresis scale mentioned above. Despite the low number of cases due to selective criteria, a statistically significant correlation (*p*-value = 0.011, OR = 4.517) was found between patients operated on between 72 h and 1 week and the postoperative worsening of paralysis, suggesting that acute onset severe motor dysfunction is an indication of acute surgery. Notably, that surpassing 24 h of complete spinal cord injury (complete loss of motor and sensory function, accompanied by autonomic impairment), the prognosis of neurological restoration is poor.

**Table 1:** Lovett’s paresis scale

Grade	Muscle effort
0	Complete paralysis
1	Visible or palpable contractions, fasciculation
2	Active movement with gravity eliminated
3	Active movement against gravity
4	Active muscle contraction against gravity with some resistance
5	Active muscle contraction against full resistance, full strenght

Furthermore, we examined the correlation between severe motor dysfunction subgroup operated on in the later acute phase (72 h to 1 week). We found a statistically significant correlation between the postoperative functional outcome and the later acute severe motor dysfunction subgroup. The more severe the motor dysfunction and the later the operation is performed, the worse the functional and symptomatic outcome (Table 2).

**Table 2:** Evolution of motor function regarding timeframes postoperatively

Operation timeframe	Postoperative motor function	No. of patients	Fisher p-value	Odds-ratio	95% CI
48 hours	Better	31	> 0.999	1.066	0.468–2.453
48 hours	Unchanged	16	0.538	0.343	0.555–3.244
48 hours	Worse	2	0.189	0.322	0.032–1.719
72 hours	Better	16	0.333	1.769	0.59–6.033
72 hours	Unchanged	6	> 0.999	0.898	0.26–2.741
72 hours	Worse	0	0.205	0.000	0.000–1.888
1 week	Better	26	0.332	0.666	0.289–1.53
1 week	Unchanged	12	0.678	0.787	0.311–1.929
1 week	Worse	8	0.013	7.141	1.333–72.21

**Fig. 3:**
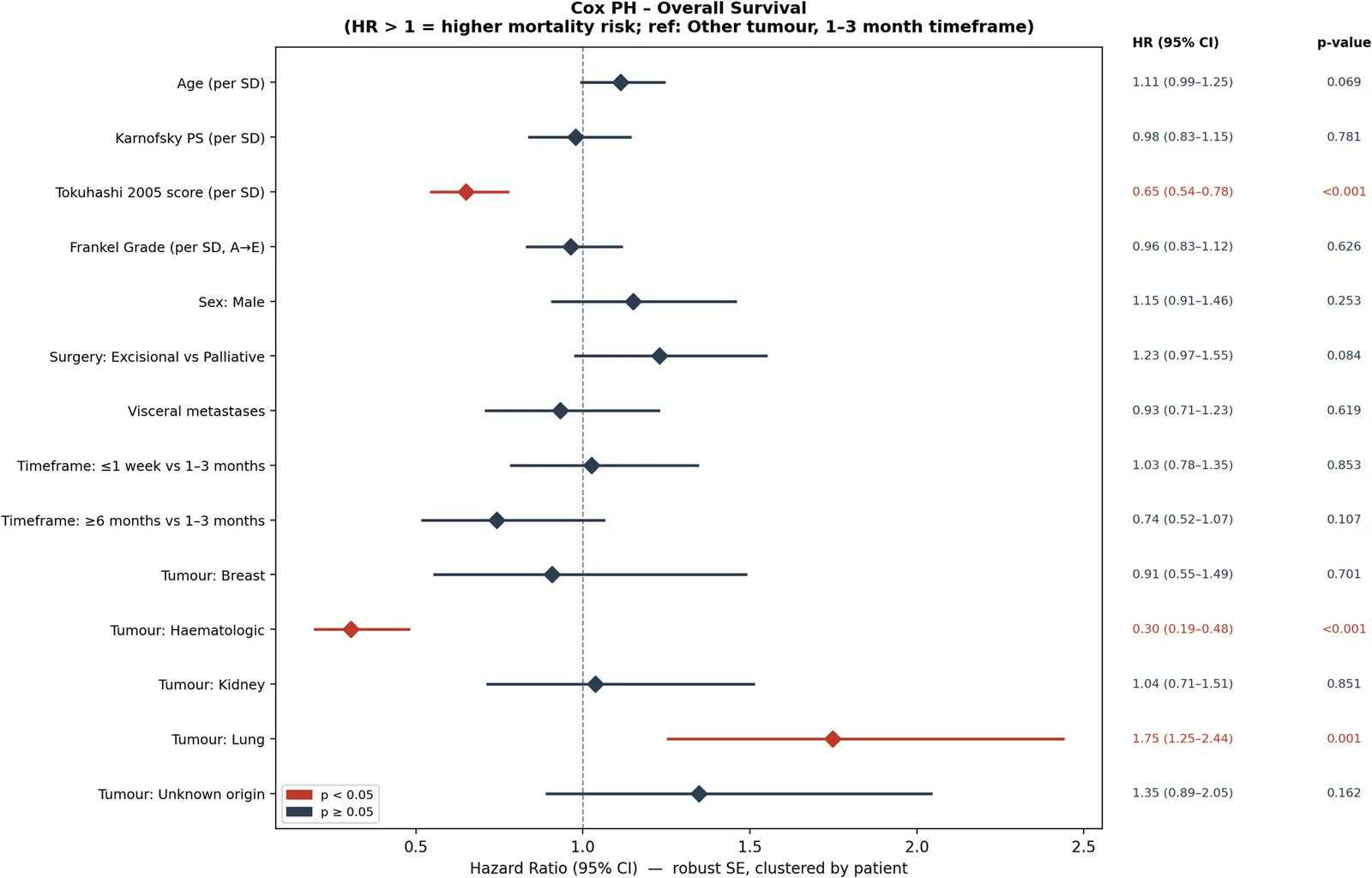
Cox proportional hazards results on postoperative survival period

To identify independent predictors of clinical outcomes, multivariable analysis was performed. Regarding overall survival, the Cox proportional hazards model revealed that obviously the Tokuhashi score was a significant predictor, confirming that patients with better prognostic scores had significantly lower mortality risk. This model also showed, that haematologic histopathology of primary tumors results in better survival rates (*p*-value = < 0.001, OR = 0.30) due to the modern targeted medical treatment modalities (Fig. 3).

**Fig. 4:**
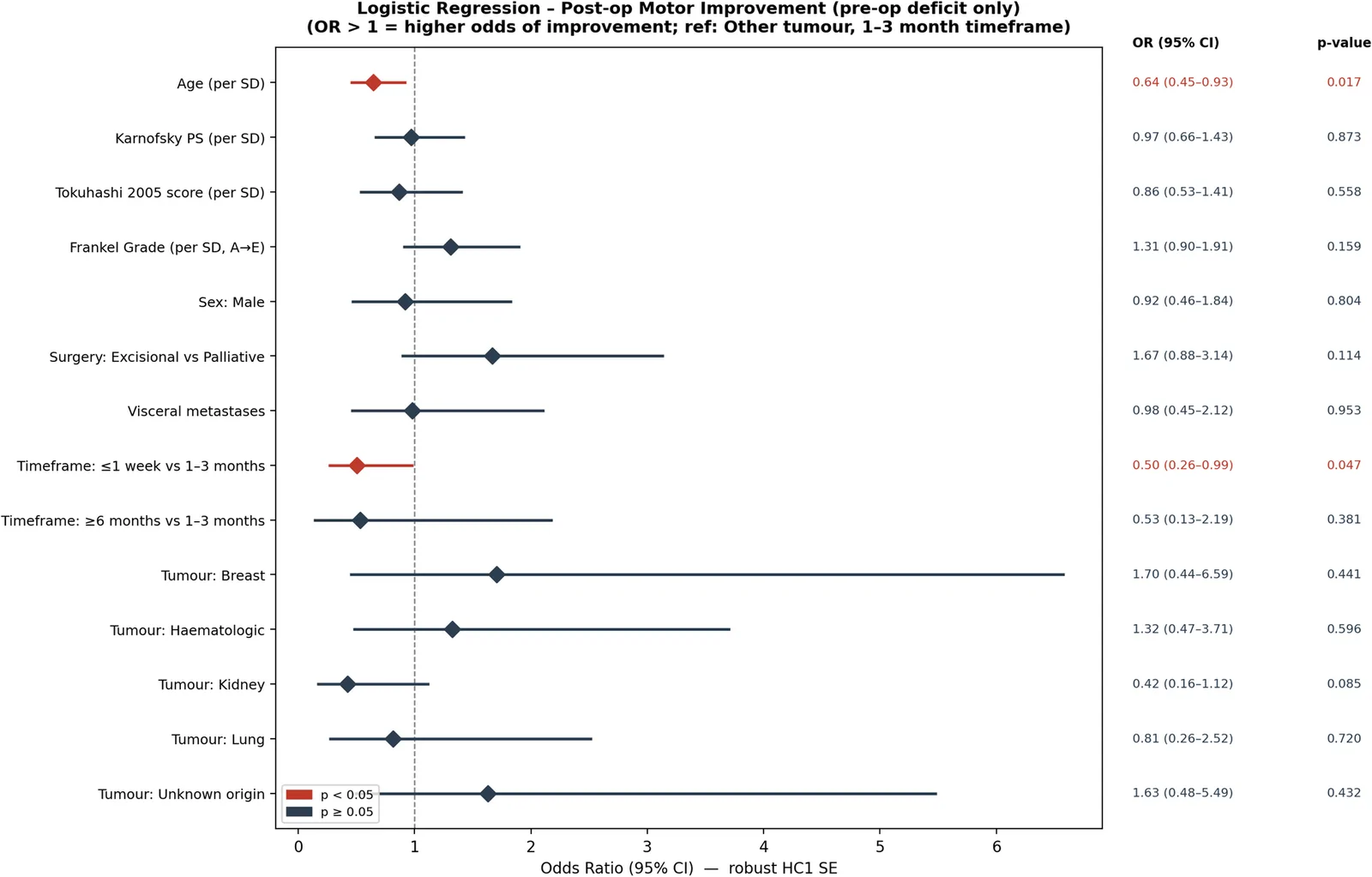
Logistic regression results on postoperative motor functions

For postoperative motor function improvement, logistic regression analysis (restricted to patients with preoperative deficits, *n* = 223) showed that younger age (*p*-value = 0.017) and the surgical timeframe were significant factors. Notably, patients operated on within 1 week had different odds of recovery compared to the 1–3 months reference group (*p*-value = 0.047, OR = 0.50), suggesting that early intervention in the chronic-subacute phase significantly influences neurological recovery independent of the primary tumor type (Fig. 4).

**Fig. 5:**
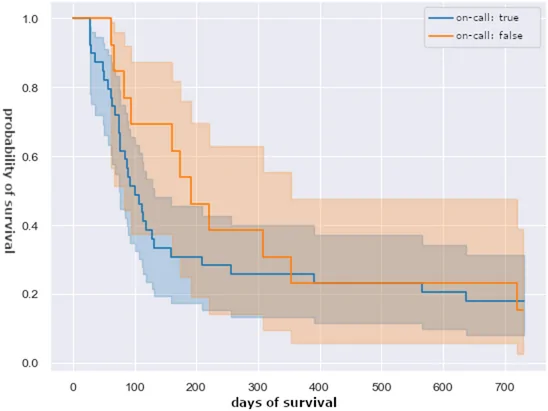
In-day timing effects on the survival period

In-day timing of the surgery was also examined in this study, one day was divided into 2 subgroups: working hours (surgery started between 8.00 and 15.00) and on-call hours (surgery started between 15.00 and 8.00 the next day). In aspects of overall postoperative symptom outcomes (pain and paresis), a significant correlation was found between the operations performed during working hours and better overall postoperative symptom outcomes, meaning better quality of life (Table 3). However, no statistically significant correlation was found between the surgeries in day timing and the overall patient *survival period* (*p*-value = 0.434; Fig. 5).

**Table 3:** In-day timing effects on overall postoperative symptom outcomes

Symptoms in total	Working hours	On-call hours	Fisher p-value	Odds-ratio	95% CI
Better	66	34	0.003	3.297	1.430–7.863
Unchanged	5	8	0.024	0.336	0.119 –0.890
Worse	9	16	0.151	0.419	0.102–1.551

## Discussion

With the increasing incidence of newly diagnosed cancer patients worldwide annually, the number of patients with vertebral metastases also increases. Because of the rapidly advancing oncological therapies, including modern biological and immunological treatments, the life expectancy of cancer patients is steadily increasing [19, 20]. As a particular oncological disease progresses, the chance of developing metastatic bone diseases (including metastatic spine tumors) increases, which portends poor prognosis for the patients [21]. Consequently, there is an increasing demand for treatments targeting symptoms caused by spinal tumors. Currently, in the era of personalized medicine, these treatments require a multidisciplinary approach, consisting of non-invasive (conservative) therapy or direct surgical intervention, in some cases supplemented with preoperative embolization of the tumor, radiotherapy, vertebroplasty, kyphoplasty [8, 10].

Rapid and accurate diagnosis of spinal metastases is crucial (history, examination, lab tests, imaging), as a few days or weeks can be decisive for optimal patient care. There is no universal accurate time of neurologic deficits becoming irreversible; in MSCC cases, several studies suggest that 48 h represents a critical threshold [22]. Conversely, other investigators report, that patients can regain ambulatory functions even if they lost their ability to walk for more than 48 h [23].

In terms of conservative treatment, in acute spinal cord compression or progressive neurological symptoms, corticosteroids are used widely, though no standardized dosing exists. Maintaining normal mean arterial pressure (MAP) is essential to ensure spinal cord perfusion and prevent vascular damage [24]. Bisphosphonates and Denosumab help prevent pathological fractures by inhibiting osteoclast activity, with Denosumab also exhibiting antitumor effects [25].

In the previous decades, the international standard treatment for spinal metastases was limited to corticosteroids and radiotherapy. Historically, in cases of MSCC, the role of surgery was not evident in managing pain or motor functions. In 2005, Patchell et al. published an article in which they described the best results in overall outcomes with surgery and postoperative radiotherapy in combination [26].

Radiotherapy is crucial in the spinal metastases treatment. It can be curative (e.g., for lymphomas), adjuvant post-surgery, or palliative for pain relief [27]. Stereotactic radiosurgery (SRS) delivers high-dose radiation to small, isolated tumors but is contraindicated in spinal cord compression. The main limitation is spinal cord radiation tolerance (45–50 Gy) [28].

Minimally invasive percutaneous vertebroplasty provides pain relief and stabilization in osteolytic metastases [29]. For highly vascularized tumors, preoperative embolization minimizes surgical bleeding and operative time, whereas in terms of non-hypervascularized tumors, there is no clear evidence of the benefit of embolization [30].

Surgery remains palliative, focusing on stabilization and decompression to preserve neurological function [7]. Posterior approaches are common, while anterior ones are used selectively. Surgical risks include bleeding, infection, and neurological deficits, depending on the localization of the disease and instrumentation failure [31]. Indications include epidural compression, instability, and radioresistant tumors, while contraindications include stable lesions and chronic paralysis. Proper patient selection requires assessing surgical risks, tumor burden, and prognosis.

The prognostic scoring systems, such as Tokuhashi [32], revised Tokuhashi [15], Tomita [16], modified Bauer [17], and Mezei [21] play a crucial role in personalized treatment planning however, none of those are perfect. These scoring systems only suggest the level of invasiveness and project the possible survival rates. They help balance the risks and benefits of surgery, radiation therapy, and supportive care based on individual patient prognosis. However, as oncology advances with targeted therapies and immunotherapies, these scoring models require continuous refinement to remain clinically relevant.

The presence of metastases means a poor prognostic factor for a cancer patient. Our study showed that of 664 patients not more than 30% (*n* = 231) exceeded the two years survival period, which we declared the censoring survival time. The median survival time is 294 days in the examined population, which can hardly be compared with the international literature date due to our selective patient criteria. Thus, it can be established that there is no significant difference in survival period between our population and the international data [33, 34] considering the conventional operation timeframes (within 48 h, within 72 h, and within 1 week). However, Lo et al. described improvement in survival data in acute timeframe (within 1 week) patients compared to later operated patients [35].

In this study surgical interventions of spinal metastases have been classified as excisional or palliative. Corpectomy, spondylectomy meaning total tumor resections with transpedicular fusion were marked as excisional, whereas dorsal decompression, subtotal tumor resection with or without fusion as palliative, and minor tumor resection for purposes of histopathologic results were marked as biopsy. Most of the studied population underwent excisional surgery (51.8%), a lower number of patients underwent palliative surgery (48.1%), while only at 0.1% of the cases biopsy was performed.

In examining the timing of acute surgical interventions, we used the 3 conventional timeframes. By evaluating these timeframes with Kaplan-Meier curves, we found that surgeries performed within 1 week from symptomatic progression did not show a significant impact on survival. However, widening the timeframes revealed significant differences in survival beyond the 1-week mark. We used Fisher’s test to confirm the probability of motor deficit presence, supporting the evidence that acute onset of severe motor deficit is an indication for acute surgery; similar results were published as well by Quraishi et al. [36], and Bresolin et al. [22].

Pain, the most prevalent symptom, can be originated from osteolytic induction of the tumor cells, causing pathologic vertebral fractures, spinal instability, and at some cases additional costo-vertebral dysfunctions, causing typically local vertebral or paravertebral pain [36]. Or either can be caused by compression of nerves, such as radicular pain [37]. In our study, we did not further classify pain into subtypes, but we analyzed its postoperative changes across all timeframes from the hyperacute phase to the chronic phase. It can be concluded that no significant correlation was found between the timing of surgery and the success of pain management, meaning that at any time of the disease, pain is a surgically sufficiently manageable symptom, supported by international literature data [38].

The second most common symptom is motor dysfunction which is clinically the most significant by highly altering the patient’s quality of life. As mentioned above, the motor dysfunction was divided into two categories: mild and severe motor dysfunction. Despite the low number of cases, we found a statistically significant correlation between patients operated on between 72 h and 1 week and postoperative worsening of paralysis. We found similar results in the available international literature [39, 40]. Although the sample size within this subgroup limits statistical power, a discernible trend indicates that patients with severe motor deficits benefit from earlier acute surgical intervention.

Our multivariable models strengthen the findings by adjusting for potential confounders such as primary tumor type and preoperative status. The significant association between the haematologic histopathological primary tumor type and survival periods showing that in spinal metastases, surgical intervention is primary palliative, survival periods mostly depend on oncological therapy.

However, the finding that surgical timing (specifically the ≤ 1 week window) independently affects motor recovery – even after adjusting logistic regression for tumor biology and Frankel grade – highlights that surgical urgency remains a critical factor in preventing permanent neurological disability.

We also examined the impact of daily timing on overall symptoms. We found diverse suggestions in the available international literature, Santangelo et al. described no significant difference in daily timing of the surgery in terms of operative time, blood loss and postoperative success [41]. Kanda et al. published an opposite opinion in their article [42].

Based on our data, it can be concluded that patients who underwent surgery during working hours had significantly better postoperative outcomes compared to those operated on during on-call hours. However, this statistical association must be interpreted with caution and should not be misconstrued as a direct causal relationship. This finding is heavily influenced by inherent case selection and baseline clinical heterogeneity. In daily neurosurgical practice, emergency procedures performed during on-call hours are predominantly reserved for patients presenting with hyperacute, severe neurological decline or spinal instability, that often possess a suboptimal baseline condition. In contrast, patients operated on during regular working hours frequently undergo a rigorous process of preoperative triage, stabilization, and interdisciplinary optimization (e.g., tumor embolization, correction of laboratory parameters, bone scintigraphy, biopsy if needed). Additionally, the optimal availability of institutional resources—such as full specialized staffing, dedicated neuro-anesthesiology teams, and comprehensive diagnostic support—during daytime hours plays a significant role in improving perioperative symptom control. Therefore, the superior outcomes observed in daytime surgeries are likely a reflection of a more favorable patient selection and comprehensive resource availability rather than the hour of the day itself.

This study focused on the timing of surgical intervention in patients with spinal metastases. A statistically significant sample size, recognized internationally, was available for analysis. As a national center, our clinic manages a diverse and heterogeneous patient population.

Our research findings, along with data from the international literature, support the need for a more precise prospective study. Considering the high patient volume facing our clinic, we aim to conduct a prospective study on the optimal timing of surgery for patients with spinal metastases, along with a comprehensive recommendation regarding surgical intervention. We have designed and prepared a large-scale, multicenter study to further investigate this topic.

### Limitations

Our study has several limitations that should be considered when interpreting the results. First, its retrospective single center design may limit the generalizability of the findings to other institutions with different surgical protocols, or patient population. Second, a degree of selection bias is inherent in the surgical timing; patients operated on within shorter timeframes may have had more acute clinical presentation or different preoperative stabilization needs, compared to those in the “later” groups. Similarly, a profound selection bias applies to the in-day timing of surgeries. Our retrospective design does not allow for complete adjustment regarding the baseline urgency, preoperative neurological severity, and acute medical status of patients operated on an emergency basis during on-call hours versus those managed electively during regular working hours. This discrepancy in patient baseline characteristics, combined with variations in nighttime resource and specialized staff availability, represents a major confounding factor when comparing working-hour and on-call outcomes.

Furthermore, although we identified key factors influencing survival and neurological outcome, the absence of extensive multivariable adjustment for all possible confounders (such as specific systemic therapies or detailed molecular markers) may affect the precision of the Hazard and Odds ratios. The heterogeneity of tumor biology – encompassing various primary cancers with different growth rates and treatment sensitivities – also introduces a layer of complexity, that is difficult to fully account for in a single cohort.

Finally, defining the exact onset of symptoms relied on patient recall and medical records, which introduces potential inaccuracy (recall bias) regarding the precise duration of neurological deficits before admission. Despite these limitations our study provides valuable real-world data on the impact of surgical timing in high-volume national neurosurgical center.

## Conclusions

Despite the poor prognosis associated with spinal metastases, timely and personalized treatment strategies can significantly improve patients’ quality of life (QoL). Surgical treatment remains a palliative therapy, aiming to give the patients the best possible QoL.

We found that conventional surgical timeframes (within 48 h, 72 h, and one week) did not show a significant impact on overall survival. The study also found that surgeries performed during working hours resulted in better postoperative outcomes compared to those conducted during on-call hours.

No significant correlation was found between the timing of surgery and pain relief, meaning pain is manageable at all timeframes. However, in the cases of motor dysfunction, a statistically significant trend suggested that early surgical intervention benefited patients with severe neurological deficits.

Acute surgical intervention remains indicated in patients with severe, rapidly progressive neurological deficits, whereas in other cases, careful preoperative optimization (e.g., tumor embolization, correction of laboratory findings, managed by a spinal specialized team) should be considered.

The findings underscore the need for a prospective, multicenter study to further explore the optimal surgical timing for spinal metastases. Given the high volume of patients treated at our institution, future research will focus on refining surgical recommendations and establishing a standardized approach to improve clinical outcomes.

## Data Availability

The datasets used and/or analyzed during the current study are available from the corresponding author upon reasonable request.

## Abbreviations

CI: Confidence interval
CT: Computed tomography
Gy: Gray (SI unit)
MAP: Mean arterial pressure
MRI: Magnetic resonance imaging
MSCC: Metastatic spinal cord compression
PET: CT-positron emission tomography-computed tomography
QoL: Quality of life
OR: Odds ratio
WHO: World Health Organization
